# A SPION-eicosane protective coating for water soluble capsules: Evidence for on-demand drug release triggered by magnetic hyperthermia

**DOI:** 10.1038/srep20271

**Published:** 2016-02-04

**Authors:** Laili Che Rose, Joseph C. Bear, Paul D. McNaughter, Paul Southern, R. Ben Piggott, Ivan P. Parkin, Sheng Qi, Andrew G. Mayes

**Affiliations:** 1 School of Chemistry, University of East Anglia, Norwich Research Park, Norwich, NR4 7TJ, UK; 2 Department of Chemistry, University College London, 20 Gordon Street, London, WC1H 0AJ, UK; 3 UCL Healthcare Biomagnetics Laboratories, Royal Institution of Great Britain, 21 Albemarle Street, London, W1S 4BS, UK; 4 Institute of Food Research, Norwich Research Park, Colney, Norwich, NR4 7UA, UK; 5 School of Pharmacy, University of East Anglia, Norwich Research Park, Norwich, NR4 7TJ, UK; 6 School of Fundamental Science, Universiti Malaysia Terengganu, 21030 Kuala Terengganu, Terengganu Darul Iman, Malaysia

## Abstract

An orally-administered system for targeted, on-demand drug delivery to the gastrointestinal (GI) tract is highly desirable due to the high instances of diseases of that organ system and harsh mechanical and physical conditions any such system has to endure. To that end, we present an iron oxide nanoparticle/wax composite capsule coating using magnetic hyperthermia as a release trigger. The coating is synthesised using a simple dip-coating process from pharmaceutically approved materials using a gelatin drug capsule as a template. We show that the coating is impervious to chemical conditions within the GI tract and is completely melted within two minutes when exposed to an RF magnetic field under biologically-relevant conditions. The overall simplicity of action, durability and non-toxic and inexpensive nature of our system demonstrated herein are key for successful drug delivery systems.

The ability to deliver drugs to specific sites of action at appropriate doses has long been a goal of medicinal and pharmaceutical scientists, due to the potential to avoid whole body side-effects, such as those associated with chemotherapeutics. Delivery to the large intestine and bowel is particularly challenging, due to the variety of aggressive environments any oral delivery system must survive before arriving at the site of action. The high and increasing incidence of bowel disease and the difficulty of delivering sufficiently high drug doses selectively to this organ make the gatrointestinal (GI) tract a crucial area for therapeutic systems development^1^,^2^,^3^,^4^,^5^. The key advantage of targeted delivery is that higher drug doses can be administered, thus increasing the chance of successful therapy whilst reducing side-effects.

Therapeutic applications for nanoparticles have grown exponentially over the last two decades, due in no small part to the developments in synthetic and analytical techniques for the preparation of nanoparticles^6^,^7^. Treatments such as magnetic fluid hyperthermia and photodynamic therapy have seen tremendous success in clinical trials for the treatment of tumours, with nanoparticulate systems at the forefront of that success^8^,^9^.

Selective targeting of such drug delivery systems has focused largely on the conjugation of cancer targeting tags (proteins, peptides, sugars) to nanoparticle surfaces and surface coatings or as part of micellar systems^10^,^11^,^12^. These have proved of limited value due to size restrictions placed on such constructions by the reticuloendothelial system, which clears foreign bodies above 20 nm in [hydrodynamic] diameter^13^. Many systems focus on the treatment of tumours via the vascular network, but as yet, “on-demand” drug delivery systems for exclusive use in the GI tract are under-developed. Current systems rely on simple pH switches or degradation through bacterial enzyme action, which can be somewhat unpredictable and offers limited control. To address this problem, we propose a drug delivery system for use in the GI tract, with potential for magnetic targeting and tracking, which can release its payload on demand using localised magnetic hyperthermia to trigger release. The system we propose is shown in Fig. 1.

Briefly, standard gelatin capsules were dip coated into a dispersion (10 wt%) of oleic acid-capped superparamagnetic iron oxide nanoparticles (SPIONs) in molten eicosane at 50 °C. This renders the capsule impervious to water/acid/base ingress and thus provides a coating that is resistant to the harsh conditions experienced in the GI tract. This was demonstrated by dissolution testing of dip-coated paracetamol-filled capsules in pH 1.2 HCl and FASSIF (fasted state simulated intestinal fluid - buffer and bile salts mimicking the intestine) at 37 °C. No drug release was observed for periods up to 24 hours under these conditions. At the desired delivery point, brief irradiation (a few minutes) from a radio frequency (RF) field with a frequency and field strength acceptable for therapeutic use^14^ causes localised magnetically-induced heating of the wax coating to a temperature just above its melting point but insufficient to cause tissue damage. The exact melting point can be tuned by mixing eicosane and docosane in various ratios to raise the melting point to just above body temperature. Melting the wax triggers fluid ingress and capsule rupture, delivering the contents into solution. Post-delivery, the ruptured capsule remnants and nanoparticles would then be excreted harmlessly in the patient’s stool.

When developing a delivery system, it is essential to consider bio-compatibility and toxicity and it is preferable only to use pharmaceutically-approved materials. This rationale informed selection of the nanoparticle synthesis method and SPION loadings, which are lower than guidelines for iron oxide contrast agents (table S1), but sufficient to cause the required response. Many other synthetic approaches provide superior control of size and polydispersity^15^,^16^, but the aqueous precipitation method chosen used only approved compounds and can be scaled easily to produce large amounts of iron oxide nanocrystals, whose size range could be controlled by varying the amount of oleic acid included in the synthesis. Analysis of the products by TEM and DLS confirmed that nanocrystals of about 10–20 nm could be synthesised reproducibly (Fig. 2a,b). Powder XRD produced patterns consistent with iron oxide (most likely magnetite Fe_3_O_4_) (Fig. 2c). SQUID measurements gave a saturation magnetization value of 59 emu g^−1^ and zero coercivity at 300 K, typical of iron oxide nanocrystals in this size regime (Fig. 2d)^17^. ATR-FTIR measurements of the purified oleic acid coated nanocrystals were difficult due to the broad-band IR absorbance of iron oxide, but the spectra were sufficient to confirm the presence of the oleic acid capping layer (Fig. S7).

Images of a coated capsule and a section showing the coating thickness are shown in Figs S3–S5. The dip-coating method used in this work, although crude, produced reproducible coating thicknesses with no defects that compromised surface protection. For these experiments, three dipping cycles were used to give an overall coating thickness of 1.5 ± 0.39 mm, and were shown to be impervious to pH and FASSIF buffer ingress using gut mimicking conditions and timescales at 37 °C (body temperature) (Fig. S6).

Heating experiments were first conducted in air by placing the dry capsule in a polystyrene weighing boat balanced on the top surface of the RF coil (Resonant circuits Limited)^18^ and monitoring using the thermal imaging camera while the capsule was irradiated at a frequency and field strength of 1 MHz and 7.5 mT respectively. Visual observation clearly showed the melting of the wax due to magnetically-induced heating in air (Fig. 3e,f). The temperature maps obtained from thermal imaging (Fig. 3a–d) show the beginning of the melting process along one edge of the capsule and an end point where the hot wax has run off the capsule surface, reaching a temperature of ~60 °C after 3 minutes irradiation. Videos showing the evolution of heat maps with time are available to view in the SI. Similar results were recorded for capsules half-submerged at the surface of 10 mL of FASSIF buffer, with dye-release observed on irradiation once the coating had melted (Fig. S2).

Temperature versus time data was extracted from the thermal image data and representative heating curves are plotted in Fig. 4. The curves displayed initial heating to a plateau value consistent with the melting point of eicosane (35–37 °C - confirmed by DSC measurements – see Fig. S1). At this point energy is required to supply the latent heat of melting, hence a plateau is observed as the material undergoes phase transition to the liquid. Once liquefied, rapid heating is observed, which tails off at high temperatures due to thermal diffusion to the external environment. Interestingly, the initial gradient of the heating curve, when the wax is still solid, is shallower than the gradient observed after melting. We interpret this as showing the difference in heating mechanisms available for nanoparticles in a constrained solid matrix (Néel heating only) compared with freely rotating particles in a dispersion (Néel and Brownian heating).

The synthesis, characterisation and activation mechanism of a magnetic hyperthermia triggered drug delivery system for on-demand drug delivery has successfully been demonstrated, and the release mechanism evaluated in aqueous systems mimicking the intestine. Integrating this system with targeting and locating devices such as magnetic field gradients and MRI is the subject of ongoing research. This is one of the only instances of the use of nanoparticles for on-demand drug release in a non-intravascular drug delivery system and has huge potential for treatment of GI diseases ranging from Crohn’s disease and ulcerative colitis to cancers.

## Materials

Iron(II) sulphate heptahydrate (≥99%), iron(III) chloride hexahydrate (97%) were purchased from Alfa Aesar. Sodium hydroxide pellets (Analytical reagent grade) and ammonium hydroxide solution (28% in water) were purchased from Fisher Scientific Limited. Oleic acid (technical grade, 90%) and eicosane (99%) were purchased from Sigma Aldrich Limited and used as received. Laboratory solvents were purchased from Sigma Aldrich Limited and were of the highest possible grade. Aqueous solutions were prepared using UHQ deionised water with a resistivity of not less than 18.2 MΩ cm^−1^.

## Methods

Iron oxide (Fe_3_O_4_) nanoparticles were synthesised using the chemical co-precipitation reaction between iron(II) and iron(III) salts in basic media in the presence of oleic acid using a slight modification of the approach described by López-López *et al.*^19^. Briefly, iron(III) chloride hexahydrate (34.0 g, 125.8 mmol) was dissolved in deionised water (175 mL) and mixed with a solution of iron(II) sulphate heptahydrate (18.0 g, 64.7 mmol) in deionised water (200 mL) in a molar ratio of 1:2 in a nitrogen purged reactor. Ammonium hydroxide solution was added under vigorous stirring in order to achieve a pH of 10, before addition of oleic acid (10 mL, 31.5 mmol). The dark brown suspension was subsequently heated to 95 °C, before cooling naturally to room temperature as soon as 95 °C was reached. The nanoparticles were precipitated by addition of 1 mol dm^−3^ HCl solution. The liquid was decanted, before isolation of the nanoparticles by centrifugation (1000 × *g*) and washing with 3 × 100 mL portions of deionised water and ethanol respectively. Finally, the precipitate was dried in a vacuum desiccator.

The nanoparticle-eicosane layer was applied to the capsules by dip-coating a gelatin capsule on the end of a smoothed glass suction tube (connected to a membrane-type vacuum pump) into an homogenised (Ultra Turrax T4, IKA, Germany) molten suspension of 10 wt.% iron oxide nanoparticles in molten eicosane (*ca.* 50 °C). A complete coating was achieved by waiting for the molten layer to cool before reversing the capsule to coat the second end with a small overlap in the centre to ensure complete coverage. The process was repeated for the addition of subsequent layers up to a total of 3 layers (1.5 mm thickness). A custom glass tube with multiple arms was constructed for dipping several capsules simultaneously.

Hyperthermia experiments were undertaken using a MACH (Magnetic Alternating Current Hyperthermia) system designed and built by Resonant Circuits Limited^18^. The 3-turn coil was 44 mm in diameter, water cooled and generated a maximum field strength of 9.2 mT at 1 MHz frequency. The temperature was monitored using a fluoroptic temperature probe (Luxtron FOT Lab Kit, Lumasense California USA). Thermal images were recorded with an Infratec (Germany) VarioCAM HR research 780 with 30 mK thermal resolution and 1280 × 960 spatial resolution. Other instrumentation details can be found in section S3.

## Additional Information

**How to cite this article**: Che Rose, L. *et al.* A SPION-eicosane protective coating for water soluble capsules: Evidence for on-demand drug release triggered by magnetic hyperthermia. *Sci. Rep.* **6**, 20271; doi: 10.1038/srep20271 (2016).

## Supplementary Material

Supplementary Information

Supplementary Information

Supplementary Information

## Figures and Tables

**Figure 1:**
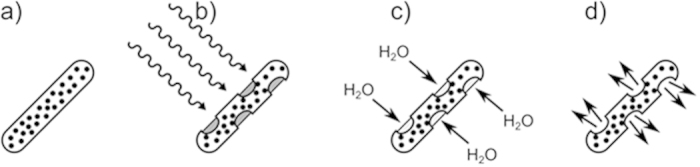
The process of capsule exposure and drug release. (**a**) An iron oxide and eicosane coated capsule, (**b**) upon exposure to radiowaves, heating occurs causing melting and formation of holes in the coating and exposure of the capsule to the surrounding environment, (**c**) fluid ingress causes capsule dissolution and (**d**) drug release.

**Figure 2:**
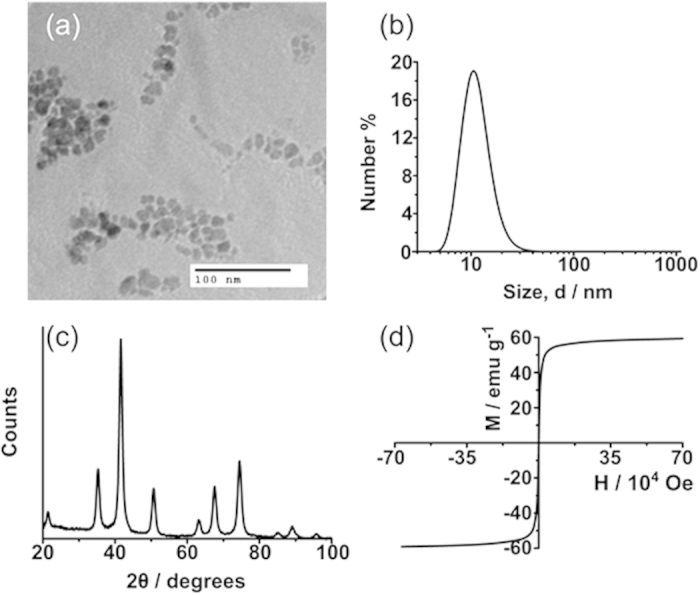
(**a**) TEM micrograph of ~10 nm iron oxide nanoparticles, (**b**) an average DLS size (by number, 10.1 nm) of the aforementioned nanoparticles, (**c**) an XRD pattern of iron oxide nanoparticles and (**d**) a SQUID magnetometry measurement showing a saturation magnetisation of 59 emu g^−1^.

**Figure 3:**
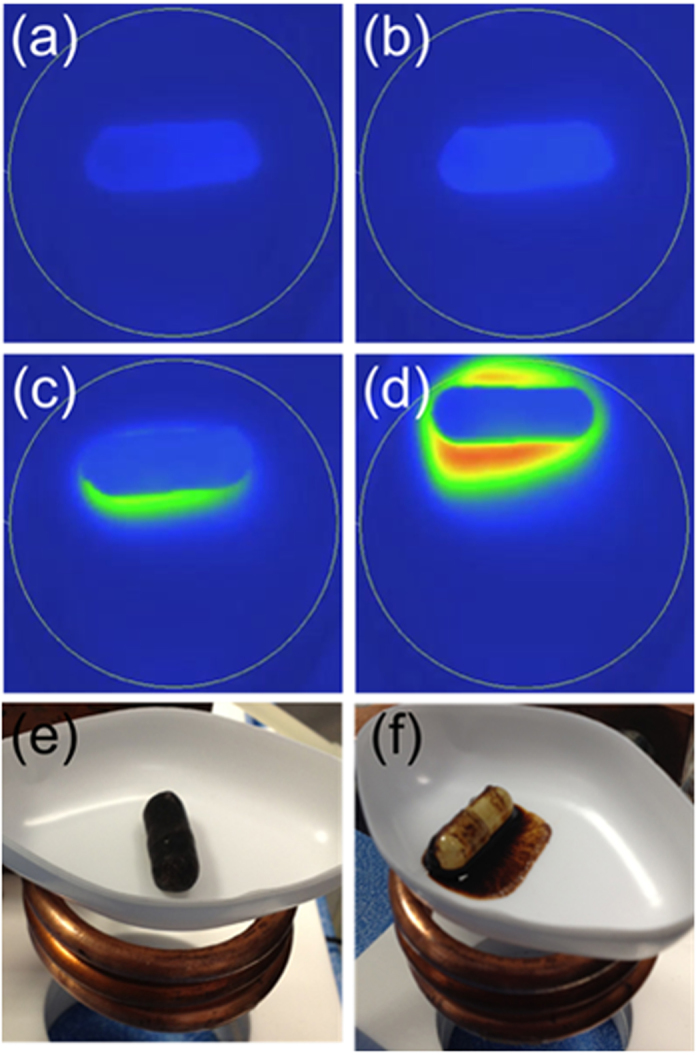
Thermal images of the coated capsules in the presence of the alternating magnetic field at time t = (a) 30 s, (temperature maximum 35.43 °C) (**b**) 90 s, (temperature maximum 37.33 °C) (**c**) 150 s, (temperature maximum 51.12 °C) and (**d**) 210 s, (temperature maximum 64.54 °C). The drawn circle surrounding the capsule indicates the position of the RF coil. Photographs of the coated capsule above the RF coil (**e**) before and (**f**) after exposure to the alternating magnetic field.

**Figure 4:**
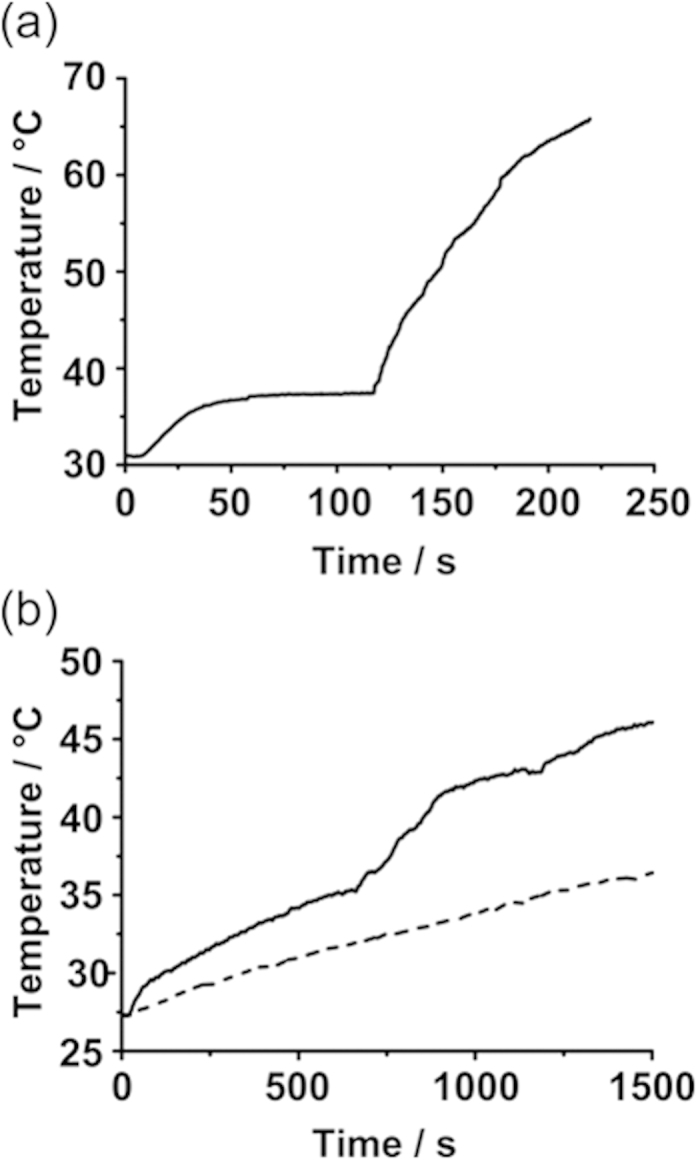
(**a**) Heating curve of 10 wt.% nanoparticles in eicosane in air and (**b**) immersed in FASSIF buffer, mimicking conditions in the gut. The capsule (solid line) shows similar heating characteristics to (**a**). The temperature of the surrounding FASSIF buffer (dashed line) also increased due to heat transfer from the melted capsule coating and the induction coil.
