# Supplementary material for: A SPION-eicosane protective coating for water soluble capsules: Evidence for on-demand drug release triggered by magnetic hyperthermia
Source: Sci Rep. 2016 Feb 4;6:20271. doi: 10.1038/srep20271 (PMC4740764; doi:10.1038/srep20271)
Supplement: Supplementary Information [file srep20271-s1.doc]

Supporting Information

A SPION-eicosane protective coating for water soluble capsules: Evidence for on-demand drug release triggered by magnetic hyperthermia

Laili Che Rose, Joseph C. Bear, Paul D. McNaughter, Paul Southern, R. Ben Piggott, Ivan P. Parkin, Sheng Qi and Andrew G. Mayes*

Table of Contents

[Figure S1:DSC curve of eicosane, showing a melting point of 40.11 °C. 2](#__RefHeading__1483_1347725236)

[Figure S2: Photographs showing dye release in FASSIF buffer from the capsule at different points during magentic hyperthermia heating experiment. 3](#__RefHeading__1485_1347725236)

[Figure S3: Photograph showing a defect-free, eicosane-iron oxide nanoparticle overcoated capsule.](#__RefHeading__1489_1347725236) 4

[Figure S4: Photograph showing a partial cross-section of an eicosane-iron oxide nanoparticle overcoated capsule, allowing observation of the coating thickness.](#__RefHeading__1491_1347725236) 5

Figure S5: Detail photograph of a single eicosane dip layer showing the smooth and uniform surface that can be achieved. …………………………………………………………………..6

Figure S6: Capsule integrity readings in (top): pH1.2 and FASSIF buffer and (bottom) in pH 1.2 and phosphate buffer pH 7.4 mimicking conditions in the GI tract……………………………7

Figure S7: FT-IR spectra for oleic acid and oleic acid-capped iron oxide NP………………...8

Table S1: Iron oxide NP loading/dosage for capsules and approved agents……………………9

[Section S1: Instrumentation](#__RefHeading__1493_1347725236)

10

Explanation of associated videos………………………………………………………….……….10


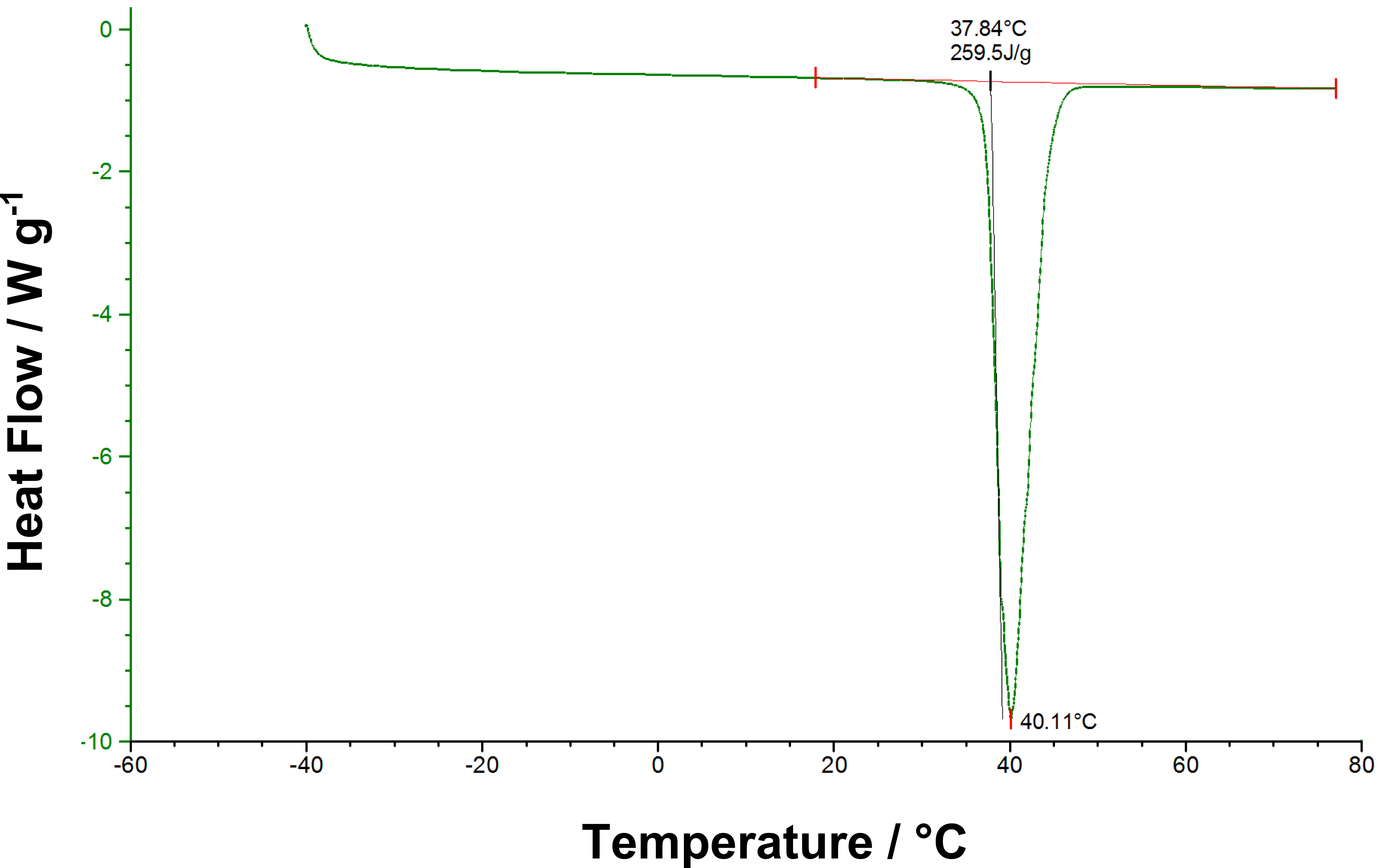


## Figure S1:DSC curve of eicosane, showing a melting point of 40.11 °C and onset of melting at about 38 °C .


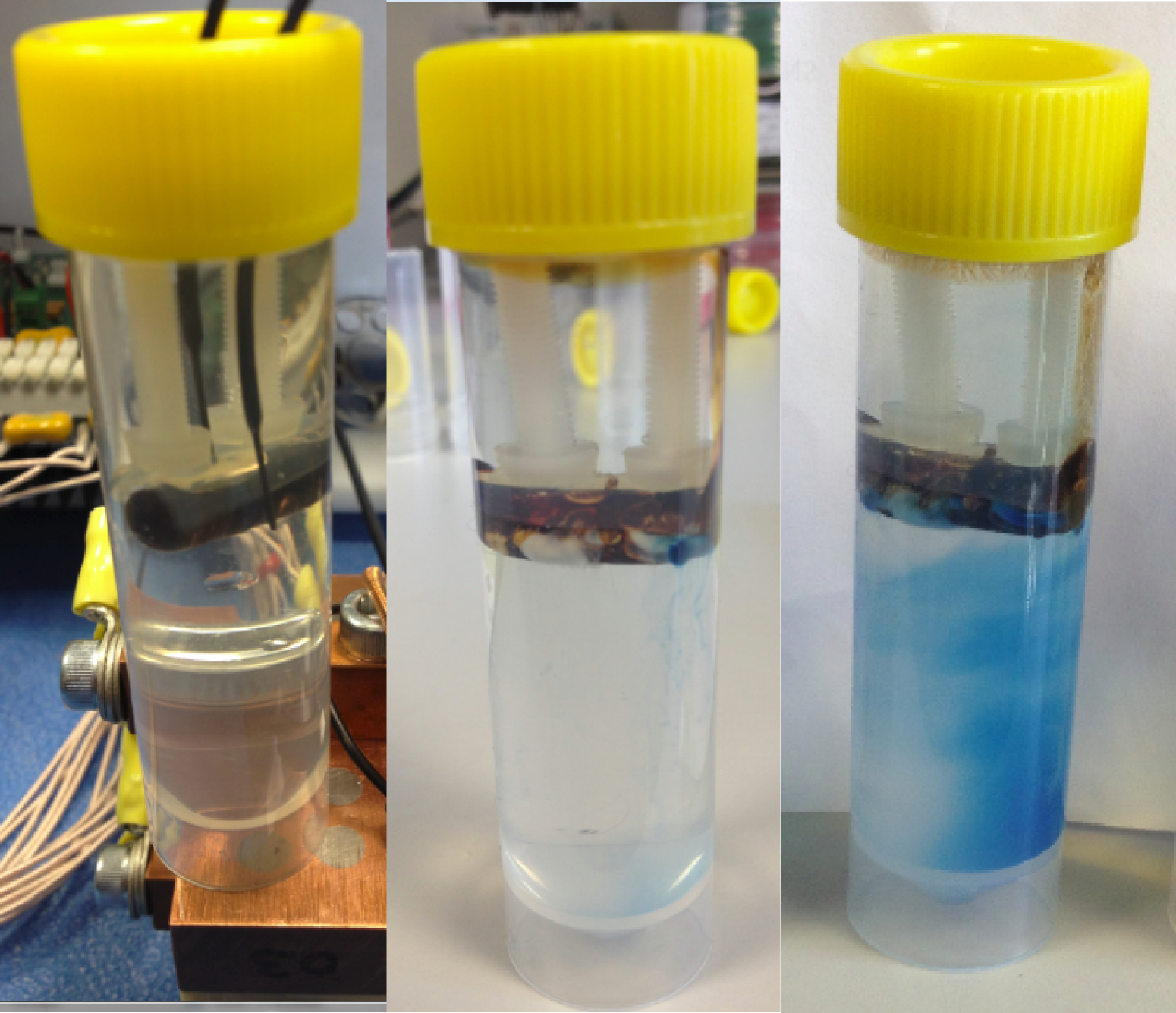


## Figure S2: Photographs showing dye release in FASSIF buffer from the capsule at different points during magentic hyperthermia heating experiment. Left at time zero, middle at the onset of dye release and right after more extensive dye release.

Detailed observation of the middle photo shows areas of bare capsule where the wax has melted and begun to flow off the capsule surface – it floats due to its lower density c.f. buffer, hence it “slides” around the capsule surface and accumulates at the top.

NOTE: there is NO brown discolouration of the buffer solution indicating NP release – only the beginning of colouration from the first trails of released dye. This observation is further confirmed in the third photo (right) where dye release is more extensive, but there is still no hint of brown colouration from NP in the buffer – colouring blue due to release of dye from the capsule. This indicates that the hydrophobic NP are strongly associated with the hydrophobic wax and do not easily disperse into the aqueous buffer, even in the presence of the bile salts that are present in FASSIF.

##
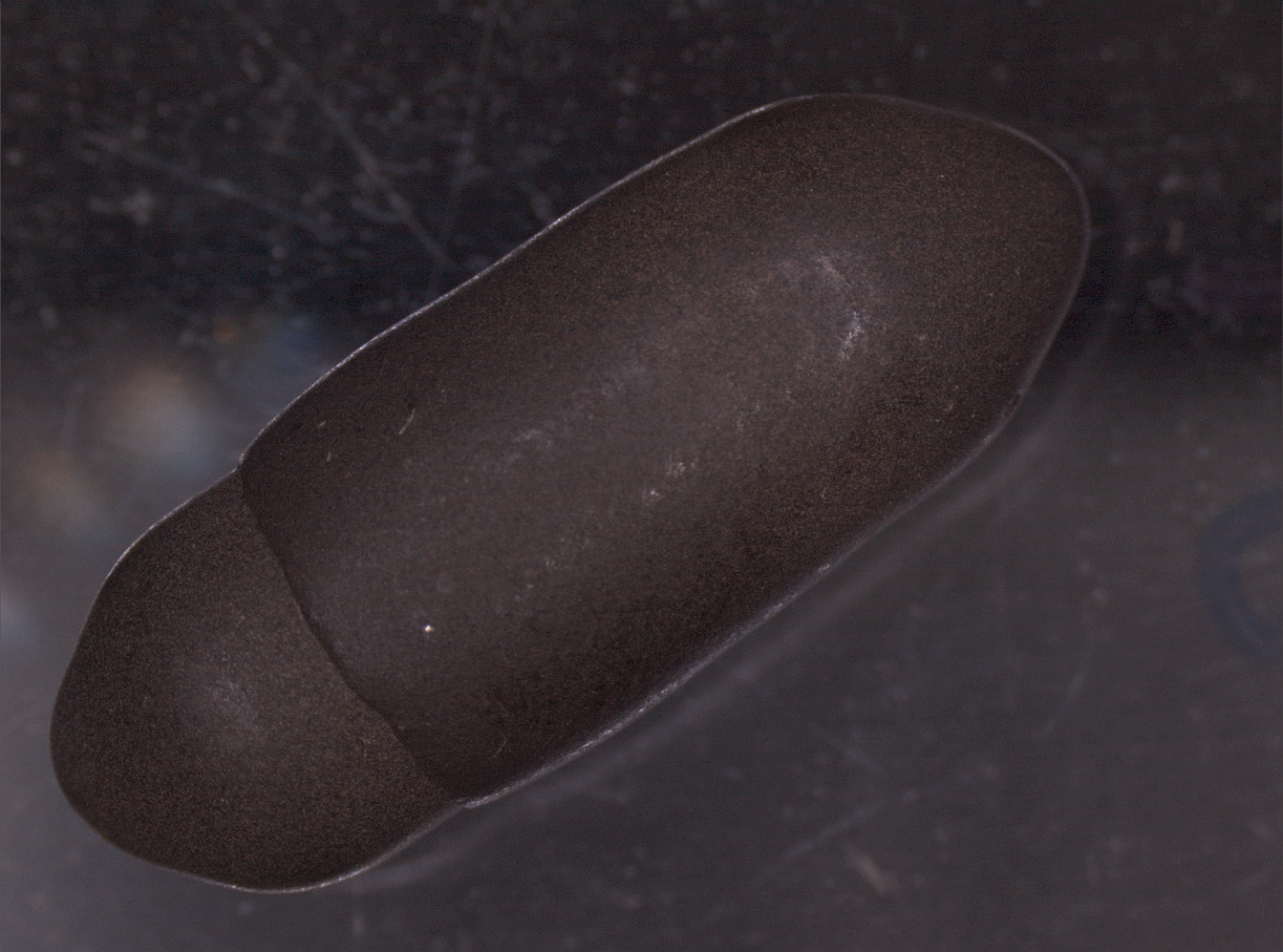


## Figure S3: Photograph showing a defect-free, eicosane-iron oxide nanoparticle overcoated capsule.


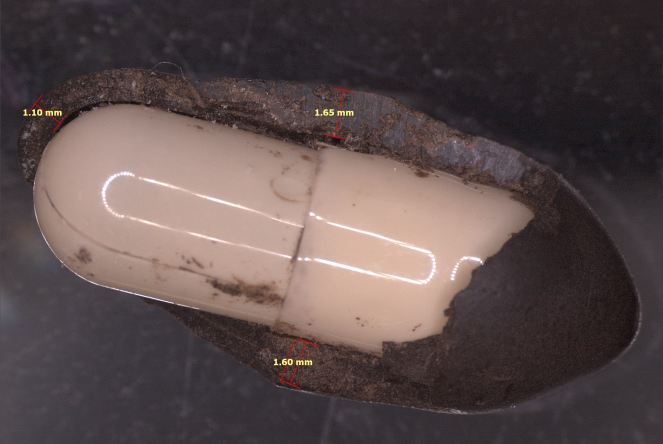


## Figure S4: Photograph showing a partial cross-section of an eicosane-iron oxide nanoparticle overcoated capsule, allowing observation of the coating thickness after 3 cycles of dipping.


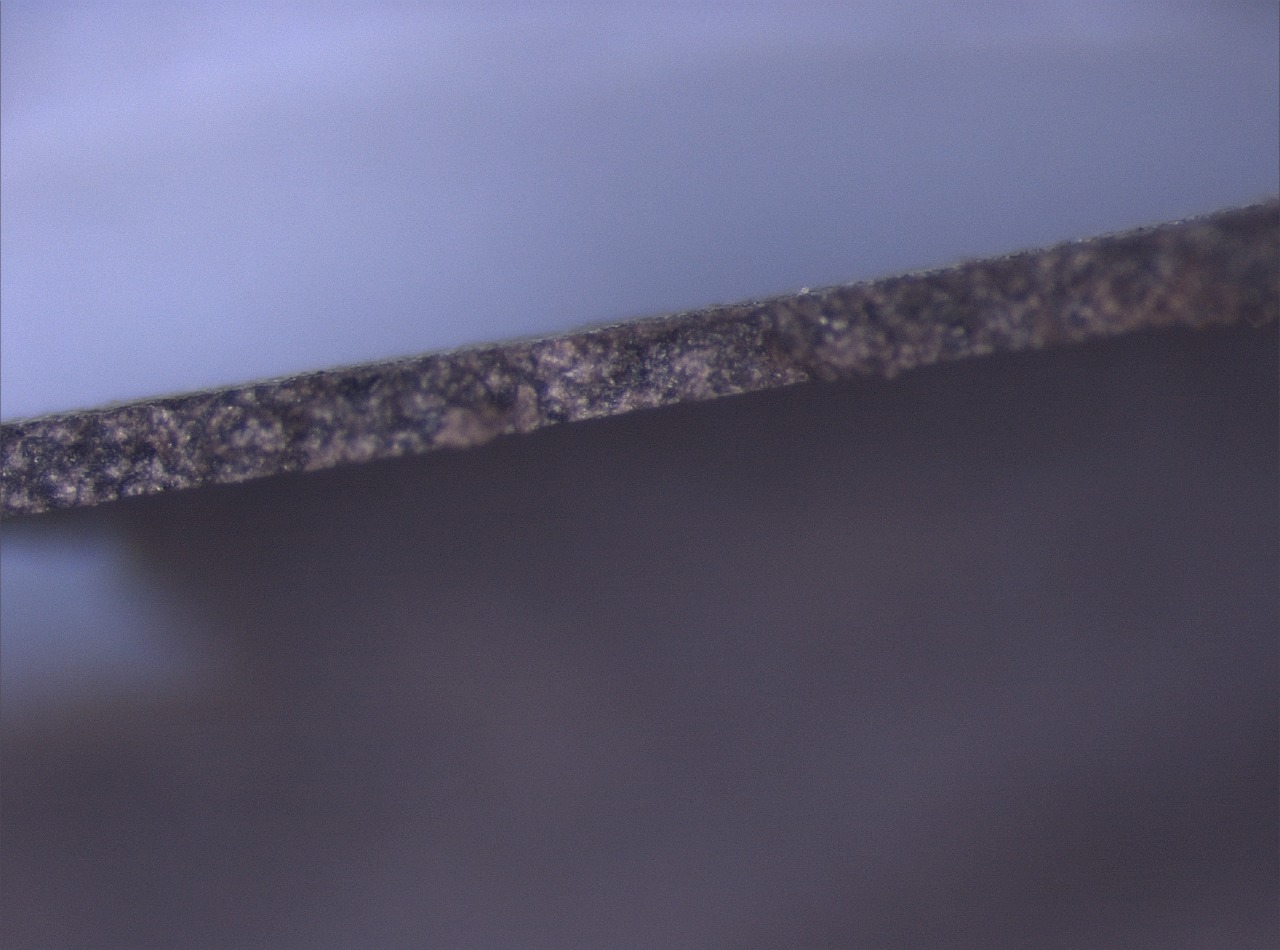


***Figure S5:*** Detail photograph of a single eicosane dip layer showing the smooth and uniform surface that can be achieved.

The effectiveness of the coating is shown in fig S6 below. For this coating material a single layer may have been sufficient, but a three layer coating was adopted in this work for the triggered release studies as insurance against defects and pinholes that might affect the integrity of the coating and cause NON-TRIGGERED release. It also allowed direct comparison with other (less effective) coatings that were tested (not reported in this work).

***
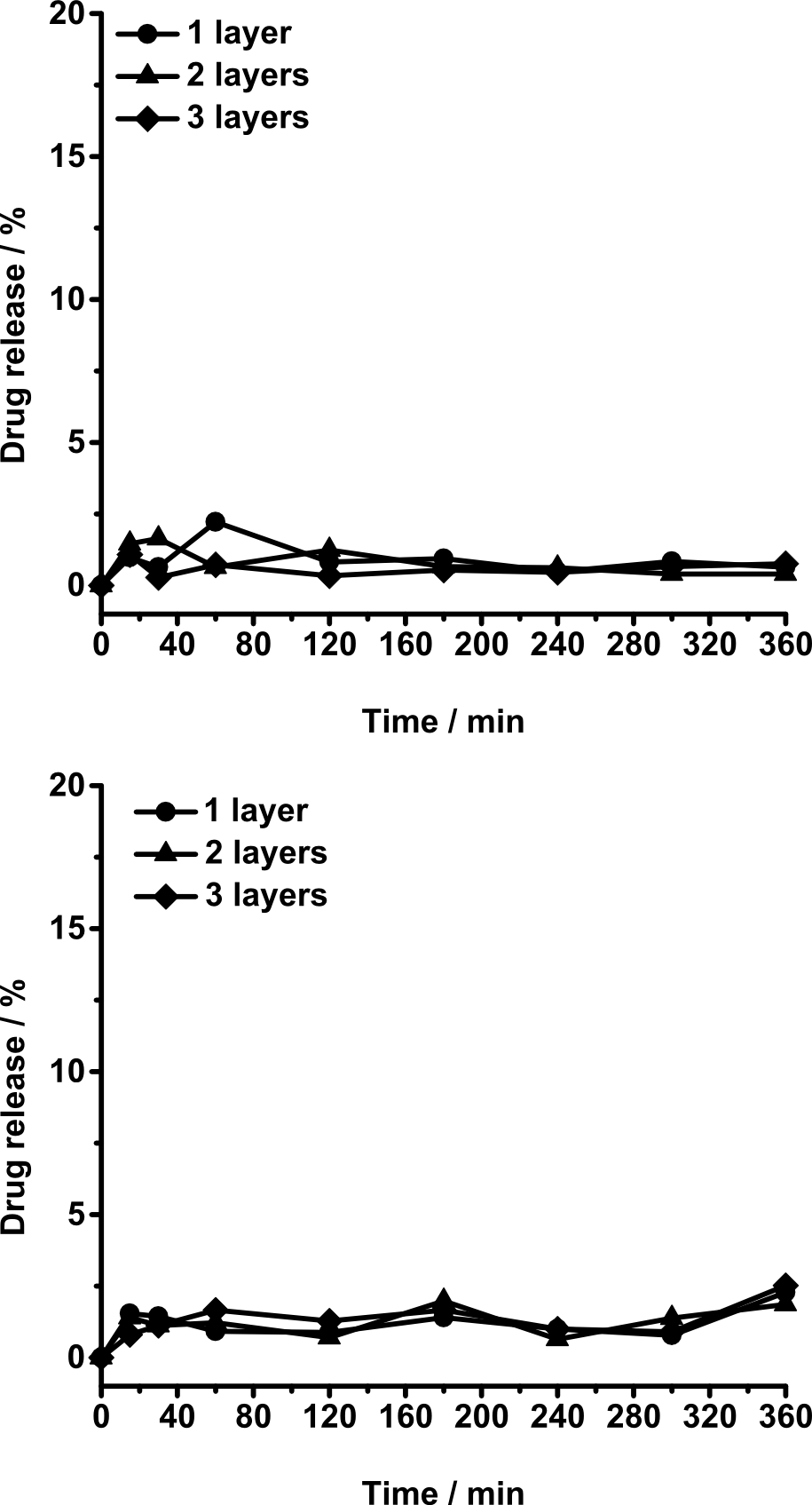
***

## Figure S6: Capsule integrity readings in (top): pH1.2 and FASSIF buffer and (bottom) in pH 1.2 and phosphate buffer pH 7.4 mimicking conditions in the GI tract. The capsule was incubated at pH 1.2 (HCl) 37°C for 2 hours to mimic stomach conditions, then switched to FASSIF or phosphate 37°C for the remaining incubation time to mimic the switch that occurs when transiting to the uppoer GI tract.

## Eicosane coatings were very robust in all conditions with virtually no drug release observed. There was little observable difference between capsules coated 1, 2 or 3 times, but the triple layer coating was adopted as insurance against any defects that might arise from the rather basic coating method adopted.

## Table S1: Nanoparticle loadings/dosage

| material | Mass of coating (mg) | average | SD | Mass Fe_3_O_4_ per dose (mg) |
| --- | --- | --- | --- | --- |
| Our capsule | | 0.1076 | 0.1104 | 0.1052 |  |  | | --- | --- | --- | --- | --- | | 0.108 | 0.003 | ~11 |
| Gastromark (600 mL dose) |  |  |  | ~150 |
| Ferridex (70 kg subject, 0.56 mg/kg) |  |  |  | ~40 |

The table shows mean and SD for the coating mass from 3 capsules coated with eicosane/NP composite (10 wt% NP). This indicates that the NP loading for a single capsule dose would be about 11 mg, compared with 40 and 150 mg for two approved iron-oxide NP-based imaging agents. It also demonstrates that, while the manual dip-coating method is relatively crude, it is also surprisingly reproducible after a little practice.

It should also be noted that the 11 mg of NP includes the oleic acid ligands. TGA analysis (data not shown) demonstrated that 20% of this mass is due to the oleic acid, so the true mass of iron oxide is nearer to 9 mg.

FT-IR spectra


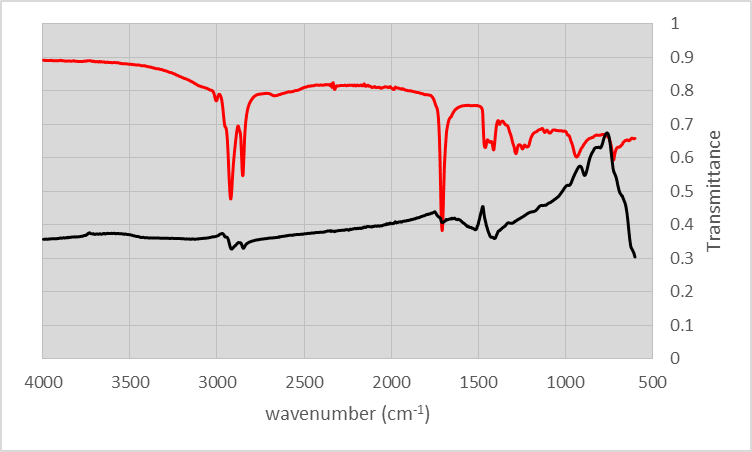


***Figure S7***: Spectra for oleic acid (red) and oleic acid stabilised iron oxide nanoparticles (black). Spectra were measured on a Bruker Alpha P FT-IR instrument with ATR attachment; 64 scans, 4 cm^-1^

Measurement of meaningful IR spectra is very difficult for iron oxide materials due to the high levels of non-specific broad band absorbance in the IR for these materials. This tends to make the already weak surface ligand signals even harder to spot, and it is almost impossible to obtain spectra that might allow interpretation of possible modes of surface interaction (e.g. from enhancement /quenching of certain modes due to surface selectivity rules). With reference to the oleic acid spectrum, the peaks in the NP spectrum can be assigned and serve to demonstrate that there are surface ligands present, which (in the presence of the reference spectrum of oleic acid) can be attributed to that ligand. The physical behaviour of the particles – not dispersible in water or buffer, but readily dispersed in hydrophobic solvents (e.g. hexane, toluene) or wax strongly indicates that the acid headgroup of oleic acid is oriented towards the NP surface with the hydrophobic tail pointing to the external environment

# Section S1: Instrumentation

Transmission electron micrographs (TEMs) were taken on a JEOL JEM 2000EX microscope at an acceleration voltage of 200 kV. Samples were drop-cast onto carbon-coated 400 mesh copper grids (Agar Scientific Limited, UK). Dynamic Light Scattering (DLS) was measured using a Malvern Zetasizer Nano-ZS. Powder X-ray diffraction (XRD) patterns were recorded on a PanAlytical diffractometer using Co Kα radiation, λ = 1.789010 Å in reflection mode. Differential Scanning Calorimetry (DSC) measurements were recorded on a TA Instruments DSC Q2000 between -40 and 80 °C. Superconducting quantum interference device (SQUID) magnetisation data was taken using a Quantum Design MPMS SQUID VSM Magnetometer (San Diego, USA) at 300 K using a field range of ±7 T. Hyperthermia experiments were undertaken using a MACH (Magnetic Alternating Current Hyperthermia) system designed and built by Resonant Circuits Limited. The 3-turn coil was 44 mm in diameter, water cooled and generated a maximum field strength of 9.2 mT at 1 MHz frequency. The temperature was monitored using a fluoroptic temperature probe (Luxtron FOT Lab Kit, Lumasense California USA). Thermal images were recorded with an Infratec (Germany) VarioCAM HR research 780 with 30mK thermal resolution and 1280x960 spatial resolution.

Associated video sequences:

Drug_release_for_eicosane_open_air (AVI format – 36 seconds run time)

This shows the image from the thermal imaging camera as a function of time for an eicosane/NP composite coated capsule sitting directly on top of the MACH system RF coil. The irradiation started at time zero and the capsule was imaged as the coating heated and melted due to hyperthermic heating. The temperature of the hottest point in the circular area around the capusule is shown in the video frames. The video clearly shows (i) gradual heating in the initial phase (ii) a plateau where the temperature does not change much as the wax undergoes a phase transition from solid to liquid and (iii) a rapid heating phase of the liquefied wax, which runs off the capsule surface and pools below it, where it continues to heat to high temperature.

Drug_release_for_eicosane_semi_immersed (AVI format run time 82 sec)

This shows the image from the thermal imaging camera as a function of time for an eicosane/NP composite coated capsule floating half-immersed in FASSIF buffer in a tube placed in the aperture of the MACH system RF coil. The level of the capsule was adjusted to be the same as that of the dry capsule sitting on top of the coil (see above). The irradiation started at time zero and the capsule was imaged as the coating heated and melted due to hyperthermic heating. The temperature of the hottest point in the circular area around the capusule is shown in the video frames. The video clearly shows (i) gradual heating in the initial phase (ii) a plateau where the temperature does not change much as the wax undergoes a phase transition from solid to liquid and (iii) a rapid heating phase of the liquefied wax, which runs off the capsule surface (mostly towards the top right of the capsue in this case) and pools below it, where it continues to heat to high temperature. The shape of the cooler “decoated” capsule can be seen as a blue shadow against the brighter background of the hotter wax below.
